# Predicting lung fibrosis in post-COVID-19 patients after discharge with follow-up chest CT findings

**DOI:** 10.1186/s43055-021-00495-0

**Published:** 2021-05-03

**Authors:** Rabab Yasin, Ahmed Abdelhakim Kamel Gomaa, Tamer Ghazy, Shaimaa Abdelhamid Hassanein, Reda Abdel latif Ibrahem, Mohamed Hossameldin Khalifa

**Affiliations:** 1 Faculty of Medicine, Menoufia University, Menofia, Egypt; 2 Giza Chest Hospital, Giza, Egypt; 3 Cardiology Department, Menoufia University, Menofia, Egypt; 4 Public Health and Community Medicine Department, Faculty of Medicine, Menoufia University, Menofia, Egypt; 5 Department of Radiology, Faculty of Medicine, University of Alexandria, Alexandria, Egypt

**Keywords:** COVID-19, Coronavirus infections, Follow-up CT, Lung fibrosis

## Abstract

**Background:**

Coronavirus disease has spread widely all over the world since the beginning of 2020, and this required rapid adequate management. High-resolution computed tomography (HRCT) has become an initial valuable tool for screening, diagnosis, and assessment of disease severity. This study aimed to assess the clinical, radiographic, and laboratory findings of COVID-19 with HRCT follow-up in discharged patients to predict lung fibrosis after COVID-19 infection in survived patients.

**Results:**

This study included two-hundred and ten patients who were tested positive for the novel coronavirus by nasopharyngeal swap, admitted to the hospital, and discharged after recovery. Patients with at least a one-time chest CT scan after discharge were enrolled. According to the presence of fibrosis on follow-up CT after discharge, patients were classified into two groups and assigned as the “non-fibrotic group” (without evident fibrosis) and “fibrotic group” (with evident fibrosis). We compared between these two groups based on the recorded clinical data, patient demographic information (i.e., sex and age), length of stay (LOS) in the hospital, admission to the ICU, laboratory results (peak C-reactive protein [CRP] level, lowest lymphocyte level, serum ferritin, high-sensitivity troponin, d-dimer, administration of steroid), and CT features (CT severity score and CT consolidation/crazy-paving score). CT score includes the CT during the hospital stay with peak opacification and follow-up CT after discharge. The average CT follow-up time after discharge is 41.5 days (range, 20 to 65 days). There was a statistically significant difference between both groups (*p* ˂0.001). Further, a multivariate analysis was performed and found that the age of the patients, initial CT severity score, consolidation/crazy-paving score, and ICU admission were independent risk factors associated with the presence of post-COVID-19 fibrosis (*p*<0.05). Chest CT severity score shows a sensitivity of 86.1%, a specificity of 78%, and an accuracy of 81.9% at a cutoff point of 10.5.

**Conclusion:**

The residual pulmonary fibrosis in COVID-19 survivors after discharge depends on many factors with the patient’s age, CT severity, consolidation/crazy-paving scores, and ICU admission as independent risk factors associated with the presence of post-COVID-19 fibrosis.

## Background

The novel coronavirus disease (COVID-19) pandemic started in December 2019 in Wuhan, China, and widely spread like wildfire across the globe. It had infected more than 61 million people and killed over 1.4 million people by December 1 as reported by the World Health Organization [1].

High-resolution computed tomography (HRCT) has become a valuable tool for screening, initial diagnosis, and assessment of disease severity [2]. The most commonly reported CT imaging findings for COVID-19 were bilateral peripheral ground-glass patches or consolidations more at basal lung segments [3–5]. Previous studies had discussed the radiological features of the disease at different stages [6–8], but radiological findings after patient discharge and during recovery need to be investigated.

The novel coronavirus SARS-CoV-2 causing COVID-19 disease is genetically similar to other strains of the coronavirus family, which are known as severe acute respiratory syndrome coronavirus (SARS-CoV) and Middle East respiratory syndrome coronavirus (MERS-CoV). All of them cause pulmonary affection and progress to acute respiratory distress syndrome (ARDS) [9]. Wu et al. [10] reported that residual pulmonary changes could be persistently found years after recovery from SARS. This raises an important question, whether similar late sequelae could also happen with COVID-19 or not.

In China, early reports indicated that 20% of COVID-19 cases have a severe course that requires hospitalization, and quarter of these hospitalized patients need intensive care admission [11]. A recent global literature survey showed that among the hospitalized patients with COVID-19, about one third of cases (33%) develop ARDS, a quarter (26%) require transfer to ICU, and 1/6 (16%) receive invasive mechanical ventilation, and for patients transferred to an ICU, nearly three quarter (75%) have ARDS [12].

Pulmonary fibrosis is a recognized sequalae of ARDS. Its pathogenesis was previously described in other coronavirus infections and was explained by viral-induced lung injury, immune response, and activation of a repair process by fibroproliferation. This repair process can result in the repair of affected lung parenchymal or may lead to pulmonary fibrosis with architectural distortion and irreversible lung changes [13]. So, pulmonary fibrotic changes occur early in the acute stage of infection as an attempt of repair following pulmonary injury. However, it is still early in the process of the disease and requires follow-up to determine if it would resolve with time or result in permanent pulmonary fibrosis [14]. The same changes occur in COVID-19 infection leading to potential increase in the risk of occurrence of pulmonary fibrosis.

Given the huge number of individuals affected by COVID-19, even a less common complication will have major health effects at the population level [15].. To date, about 28 million people have recovered from COVID-19 worldwide after more than 10 months from the start of the pandemic; more concerns about long-term lung changes following infection have evolved, yet no sufficient data is available for COVID-19 patients after discharge. So, it is important to start identifying the possibility of development of pulmonary fibrosis in the survivor population after recovery

The aim of our study is to assess the clinical, radiographic, and laboratory findings of COVID-19 with HRCT follow-up in discharged patients to predict lung fibrosis after COVID-19 infection.

## Methods

This study included two-hundred and ten patients who were tested positive for the novel coronavirus by nasopharyngeal swap, admitted to the hospital, and discharged after recovery. Patients with at least one-time chest CT scan during hospital stay and another follow-up CT after discharge were enrolled in our retrospective study in the period of 1st of August to 1st of December 2020.

The inclusion criteria included the following: (1) confirmed COVID-19 cases by nasopharyngeal swab RT-PCR testing, (2) hospitalized patients, and (3) patients who underwent initial CT during hospitalization and follow-up CT after discharge.

The discharge criterion matched the following conditions: (1) no fever for more than 3 days, (2) relief of dyspnea, (3) improvement in radiological abnormalities on chest X-ray or CT, and (4) two consecutive negative COVID-19 nucleic acid detection at least 24 h apart [16].

There were 149 males and 61 females with male to female distribution of 2.4:1; their age ranged from 18 to 94 years old with a mean age of 53.85±14.84.

The study protocol was approved by the local ethics committee. All patients provided a written informed consent.

According to the presence of fibrosis on follow-up CT after discharge, patients were classified into two groups and assigned as the “non-fibrotic group” (without evident fibrosis) and “fibrotic group” (with evident fibrosis).

We compared between these two groups based on the recorded clinical data, patient demographic information (i.e., sex and age), length of stay (LOS) in the hospital, admission to the ICU, laboratory results (peak C-reactive protein [CRP] level, lowest lymphocyte level, serum ferritin, high-sensitivity troponin, d-dimer, administration of steroid), and CT features (CT severity score and CT consolidation/crazy-paving score). CT score includes the CT during the hospital stay with peak opacification and follow-up CT after discharge. The average CT follow-up time after discharge is 41.5 days (range, 20 to 65 days).

### Image acquisition

All CT examination was performed using two multidetector CT scanner (Somatom Perspective, Siemens, Germany and Optima CT 540, GE, USA), using the following parameters: tube voltage = 120 kVp, tube current (regulated by automatic dose modulation) 30–75 mAs, pitch=1–1.25mm, matrix = 512 × 512, slice thickness = 5 mm, and FOV = 350 mm × 350 mm.

The patients were in the supine and headfirst position and received scanning with breath held. No contrast was administered. All images were transmitted to the post-processing workstation and reconstructed using high-resolution and conventional algorithms at a slice thickness of 1–1.25 mm.

Three experienced radiologists (20 and15 years of clinical experience in chest imaging) reviewed all the scans; they were blinded to the patients’ clinical and laboratory data. The final decisions were established by consensus.

### Image analysis

The initial chest CT was reviewed for each patient, and the CT severity score was estimated for each one of the five lung lobes by calculation of the dissemination of the chest manifestations (opacity), namely the ground-glass opacity (GGO), consolidation, crazy-paving pattern, septal thickening, and pulmonary fibrosis giving score (0–4) for 0, 25, 50, and ≥75% involvement, respectively, with the sum representing the total severity scores for the whole lung (0–20). If a patient had multiple CT examinations during hospitalization, the most severe CT examination during the disease progression was scored.

Previous studies [17, 18] reported that the degree of consolidation and crazy-paving pattern were highly suggestive for the disease progression/peak, so we used the total sum extent of crazy paving and consolidation as an indicator for the disease severity. The severity score for the consolidation and crazy paving was calculated for each lobe using the same criteria (0–4 scores), and the total score for the lungs is the sum of individual lobes (0–20 scores).

Ground-glass opacity (GGO) is defined as an increase in the lung density, but the bronchial vascular bundles are still visible. Consolidation is defined as opacification in which the underlying vasculature was obscured. Fibrosis was defined as parenchymal bands, irregular interfaces (bronchovascular, pleural, or mediastinal), coarse reticular pattern, and traction bronchiectasis [19].

### Statistical analysis

The data were collected and analyzed by Statistical Package for Social Science (SPSS) version 17.0 on an IBM-compatible computer (SPSS Inc., Chicago, IL, USA). The qualitative data was described as number and percentage and analyzed by using the chi-square test. Quantitative data was described as mean, standard deviation, and range; the *t* test and Mann-Whitney *U* test were used to compare normally and not normally distributed quantitative data, respectively.

Binary logistic regression was used to predict the independent risk factors of fibrosis after COVID-19 recovery. The receiver operating characteristic (ROC) curve was used to estimate the accuracy of fibrosis prediction by chest CT severity score. A *p* value of less than 0.05 was considered significant.

## Results

This study included two-hundred and ten patients who were tested positive for the novel coronavirus by nasopharyngeal swap, admitted to the hospital, and discharged after recovery. Patients with at least one-time chest CT scan after discharge were enrolled in our retrospective study in the period of 1 August to 1 December 2020.

There were 149 males and 61 females with a male to female distribution of 2.4:1; their age ranged from 18 to 94 years old with a mean age of 53.85±14.84.

Table 1 shows the general characteristics of the studied patients, including patient demographic information, clinical information (admission to the ICU, administration of steroid, and length of stay (LOS) in the hospital), and laboratory studies including the lowest lymphocyte level, C-reactive protein (CRP) level, serum ferritin, high-sensitivity troponin, d-dimer, and radiological findings (CT severity score and CT consolidation/crazy-paving score).

**Table 1:** General table characteristics of the studied patients

	The studied group, *N* = 210
**Age (years)**	
Mean ±SD	53.85±14.84
Range	18–94
**Sex**	
Male	149 (71.0%)
Female	61 (29.0%)
**Severity score**	
Mean ±SD	11.23±5.07
Range	1–20
**Consolidation/crazy-paving score**	
Mean ±SD	8.89±5.30
Range	0–20
**Length of hospital stay**	
Mean ±SD	15.63±16.99
Range	1–170
**ICU admission**	
No	158 (75.2%)
Yes	52 (24.8%)
**Steroid**	
No	34 (16.2%)
Yes	176 (83.8%)
**High-sensitivity troponin**	
Normal	165 (78.6%)
High	45 (21.4%)
**Ferritin**	
Normal	67 (31.9%)
High	143 (68.1%)
**Fibrosis on follow-up CT**	
No	109 (51.9%)
Yes	101 (48.1%)

Regarding the peak CT manifestations of COVID-19 pneumonia, it showed bilateral and peripheral distributions in 143 patients (68.1%) with the GGO and crazy-paving appearance as the predominant pattern (153 patients, 72.9%) followed by consolidation (134 patients, 63.8 %) and air bronchogram as the common findings (121 patients, 57.6%) while fibrosis was seen in 86 patients (41%) and pleural effusion was seen only in 21 patients (1.0%).

According to the presence of fibrosis on follow-up CT after discharge, patients were classified into two groups and assigned as the “non-fibrotic group” (without evident fibrosis) and “fibrotic group” (with evident fibrosis).

Table 2 shows the CT characteristic features of the initial/peak CT in both fibrotic and non-fibrotic groups; the number of affected segments was significantly higher in the fibrotic group (*p*<0.001).

**Table 2:** Comparison of particular characteristics between the groups on peak CT imaging

Characteristic		Non-fibrotic group (109), *N* (%)	Fibrotic group (101), *N* (%)		*p* value
**Number of affected segments**		9.69 ± 3.13 5–16	15.12 ± 2.91 8–22	*t* test 13.0	<0.001
**Location**	Upper lobe	53 (48.6%)	45 (44.6%)	*Z* test 0.45	0.65
	Middle lobe or lingula	78 (71.6%)	80 (79.2%)	*Z* test 1.12	0.26
	Lower lobe	85 (78.0%)	87 (86.1%)	*Z* test 1.35	0.18
**Distribution**	Central	10 (9.2%)	9 (8.9%)	*Z* test 0.17	0.86
	Peripheral	65 (59.6%)	74 (73.3%)	*Z* test 1.94	0.05
	Central and peripheral	34 (31.2%)	28 (27.7%)	*Z* test 0.40	0.69
**Opacification**	Pure GGO	56 (51.4%)	20 (19.8%)	*Z* test 4.61	<0.001
	GGO with consolidation	30 (27.5%)	47 (46.5%)	*Z* test 2.71	0.007
	Pure consolidation	23 (21.1%)	34 (33.7%)	*Z* test 1.89	0.06
	Bronchiectasis	3 (2.8%)	8 (7.9%)	*Z* test 1.37	0.17
	Crazy paving	66 (60.6%)	87 (86.1%)	*Z* test 4.01	<0.001
	Air bronchogram	48 (44.0%)	73 (72.3%)	*Z* test 4.0	<0.001
	Fibrosis with irregular interface, coarse reticular pattern, and parenchymal band	23 (21.1%)	63 (62.4%)	*Z* test 5.94	<0.001
	Pleural effusion	8 (7.3%)	13 (12.9%)	*Z* test 1.10	0.27

**Fig. 1:**
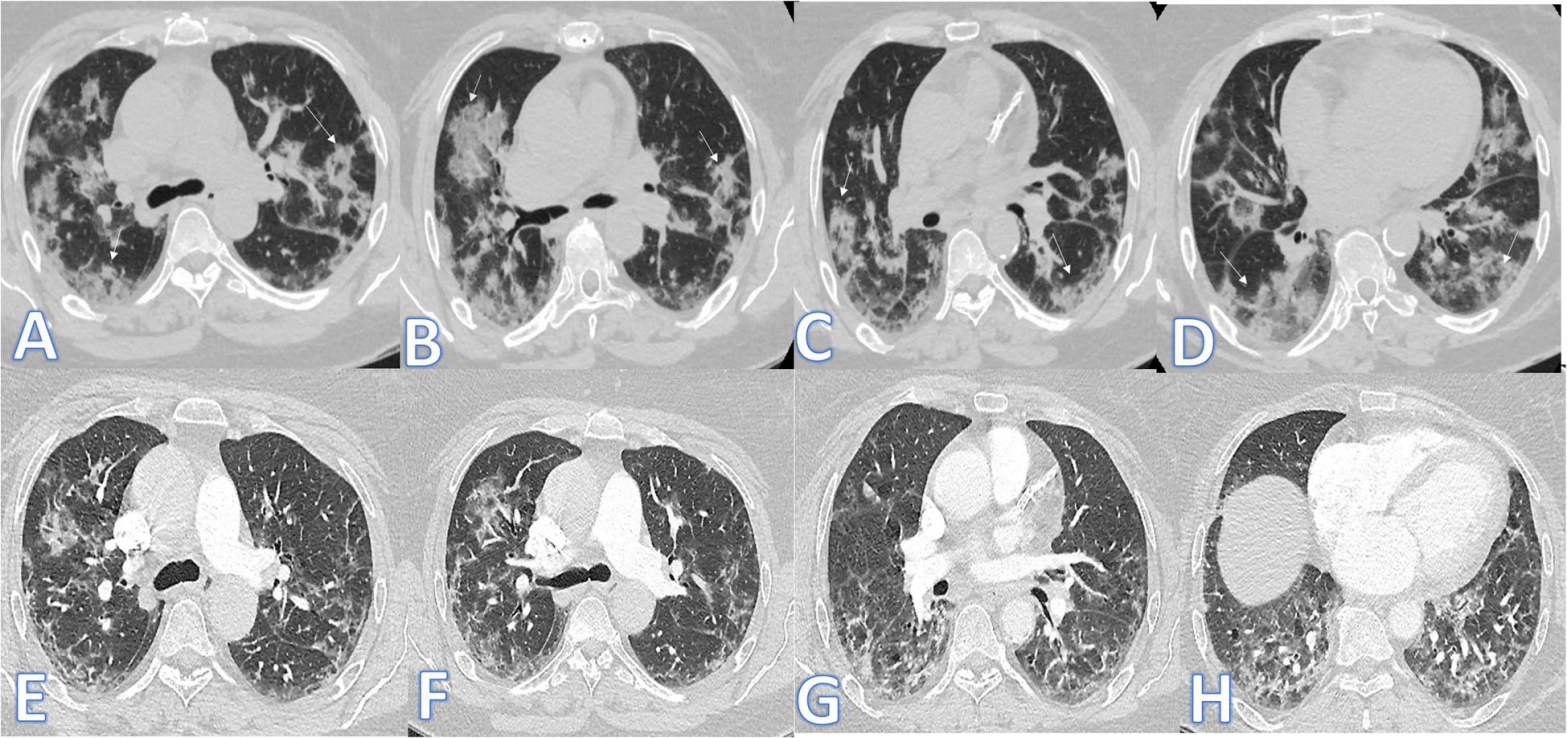
Fifty-five-year-old female: she has DM, HTN, and IHD; peak CT severity score 12; consolidation/crazy-paving score 10. She was admitted to the ICU; laboratory results show lymphopenia, high CRP, d-dimer, serum ferritin, and high-sensitivity troponin. Steroid was given; length of stay during hospitalization is 15 days. Peak CT during admission (**a**–**d**) showed bilateral consolidation patches more at the lower lobes with a crazy-paving appearance (arrows). Follow-up CT 3 weeks after discharge (**e**–**h**) shows lung fibrosis with a coarse reticular pattern mainly dependent (arrows).

**Fig. 2:**
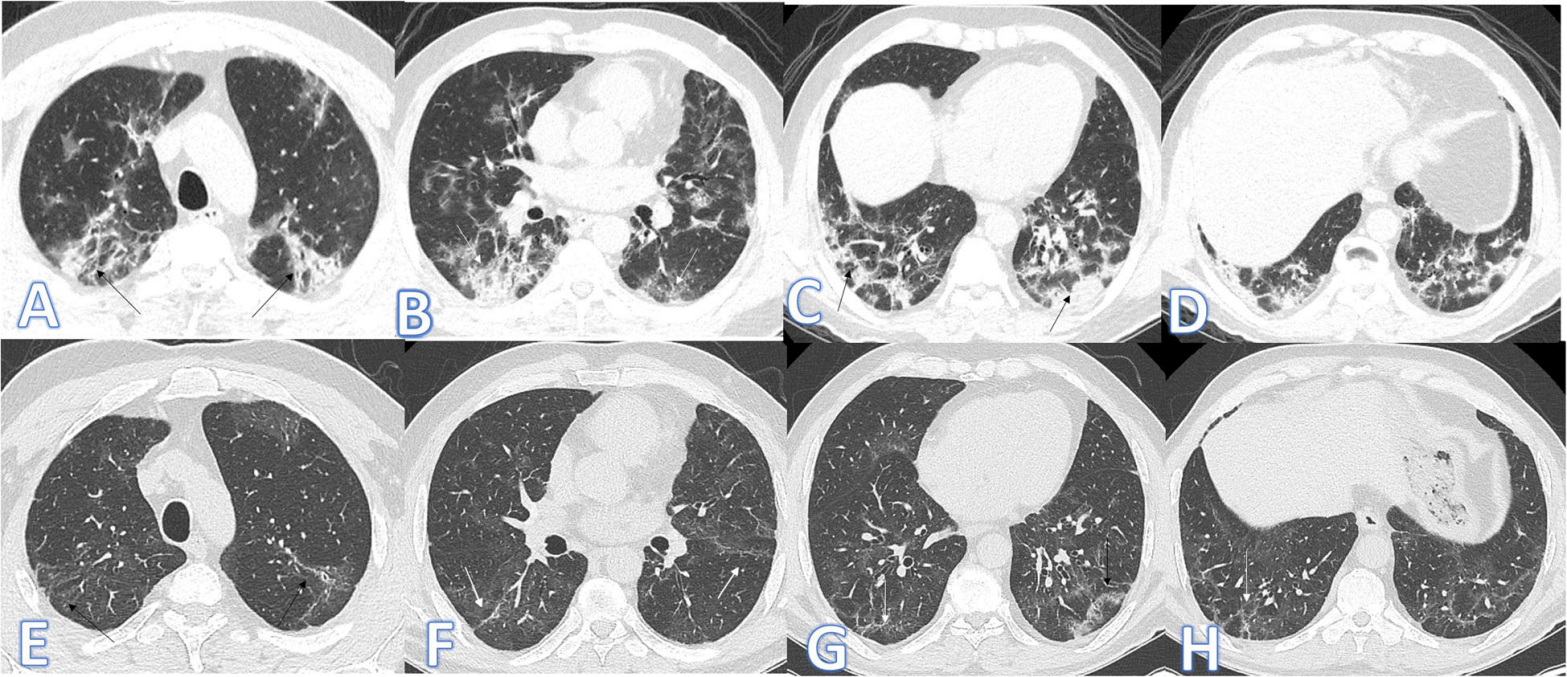
Fifty-nine-year-old male: he has DM; peak CT severity score 10; consolidation/crazy-paving score 8. No ICU admission; laboratory results show lymphopenia, high CRP, d-dimer, and serum ferritin. Steroid was given; length of stay during hospitalization is 10 days. Peak CT (**a**–**d**) shows bilateral peripheral consolidation patches mainly in the lower lobes (arrows). Follow-up CT 2 months after discharge (**e**–**h**) shows residual fibrosis with parenchymal bands, coarse reticular pattern (arrows), and atoll sign (black arrow in **g**).

**Fig. 3:**
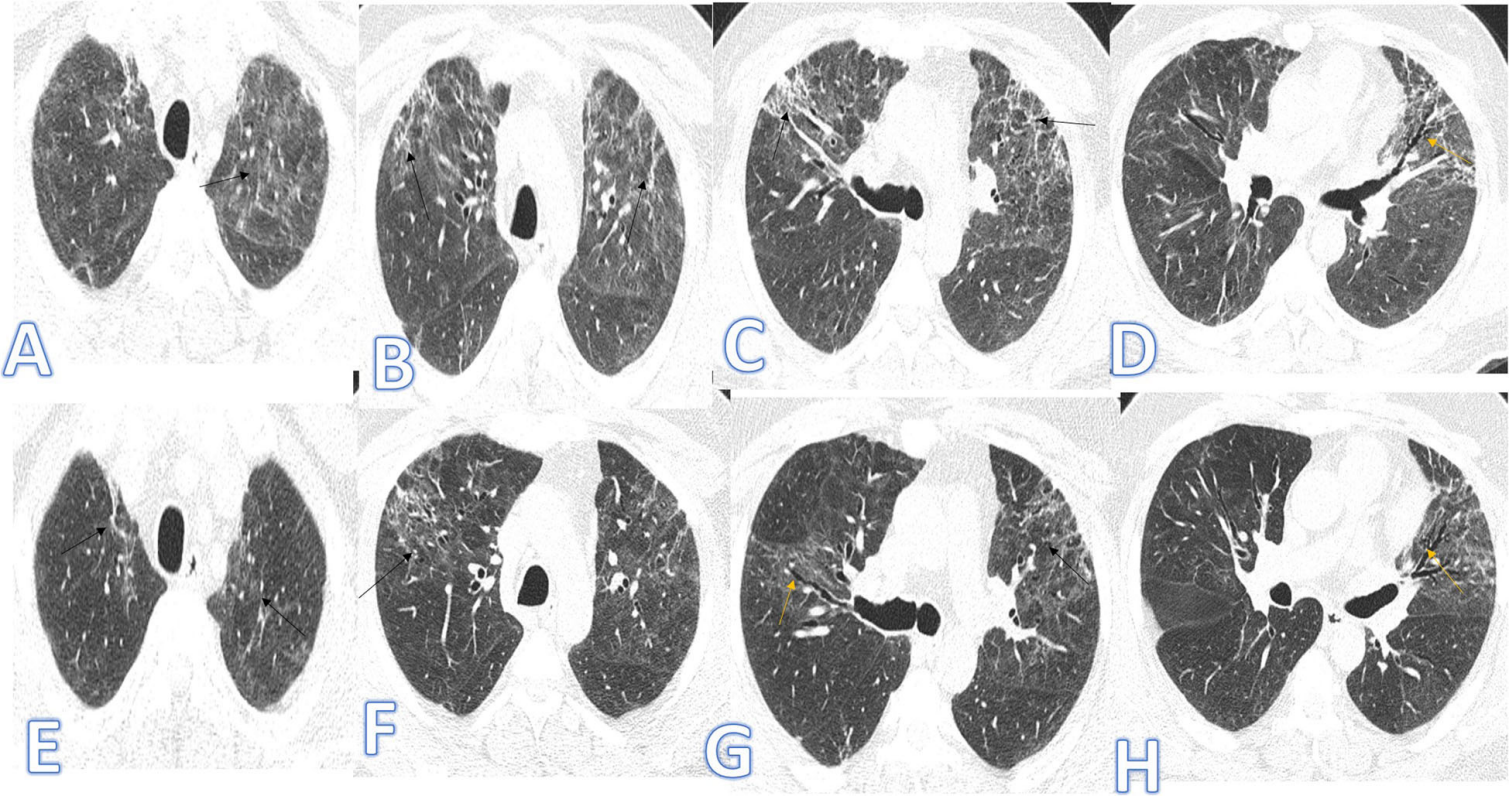
Forty-three-year-old male, he has no past medical illness; peak CT severity score 18; consolidation/crazy-paving score 14. He was admitted to the ICU; laboratory results showed lymphopenia, high CRP, d-dimer, and serum ferritin. Steroid was given; length of stay during hospitalization is 65 days. CT after 3 weeks of admission (**a**–**d**) showed bilateral GGO with bilateral upper lobe fibrotic bands (arrows) and traction bronchiectasis (orange arrows). Follow-up CT 2 months after discharge (**e**–**h**) showed lung fibrosis with parenchymal bands, coarse reticular pattern (arrows), and mild traction bronchiectasis at the anterior segments of both upper lobes (orange arrows).

There were no significant differences between both groups as regards the distribution and location of the lesions. Pure GGO was statistically higher in the non-fibrotic group, while pure consolidation or GGO with consolidation (Figs. 1 and 2), crazy paving, air bronchogram, and fibrotic changes (Fig. 3) were significantly higher in fibrotic groups (*p*<0.001).

**Fig. 4:**
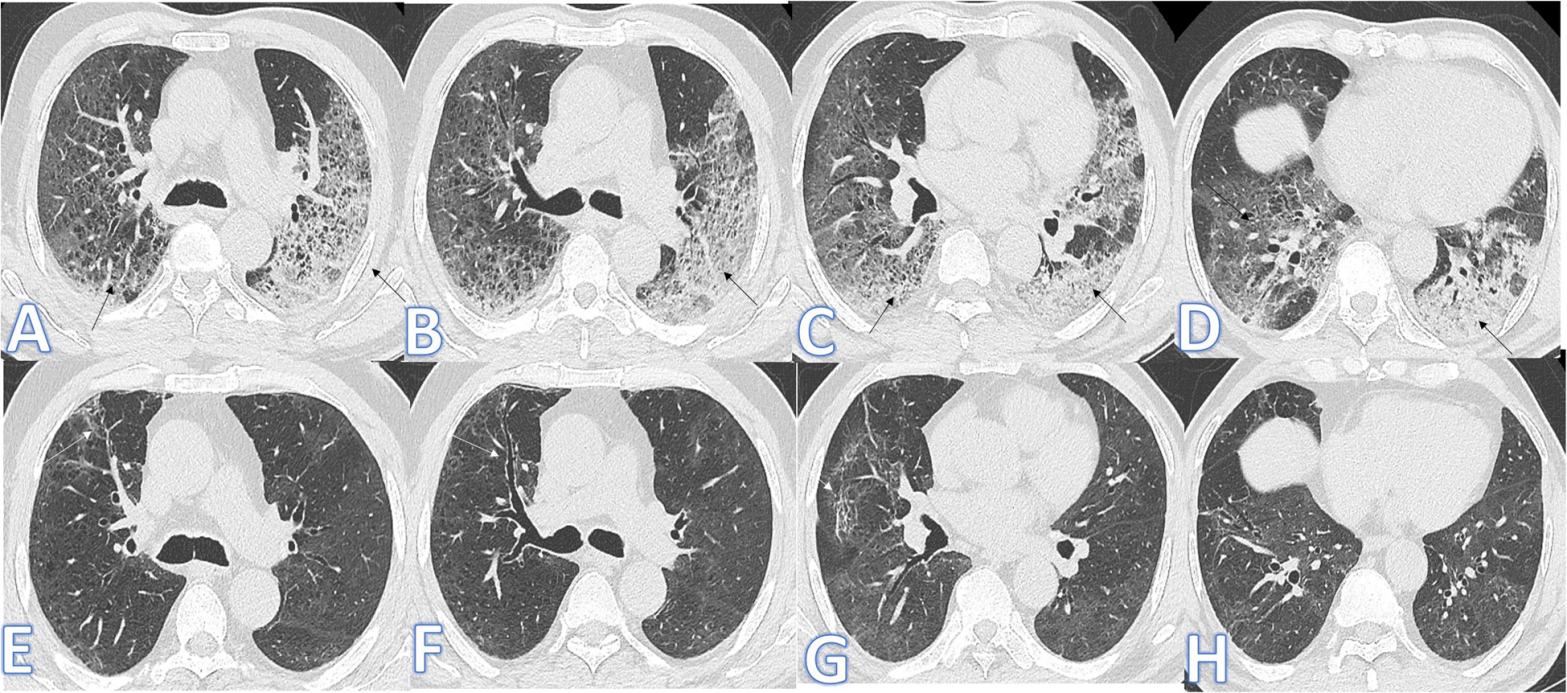
Sixty-four-year-old male: he has DM and HTN; peak CT severity score 17; consolidation/crazy-paving score 11. No ICU admission; laboratory results showed lymphopenia, high CRP, d-dimer, and serum ferritin. Steroid was given; length of stay during hospitalization is 20 days. Peak CT (**a**–**d**) showed GGO with interstitial thickening giving a crazy-paving appearance (arrows). Follow-up CT 45days after discharge (**e**–**h**) showed almost resolution of the previous with residual fibrosis with parenchymal bands, coarse reticular pattern, and mild traction bronchiectasis at the anterior segment of the right upper lobe and lateral segment of the right middle lobe (arrows)

**Fig. 5:**
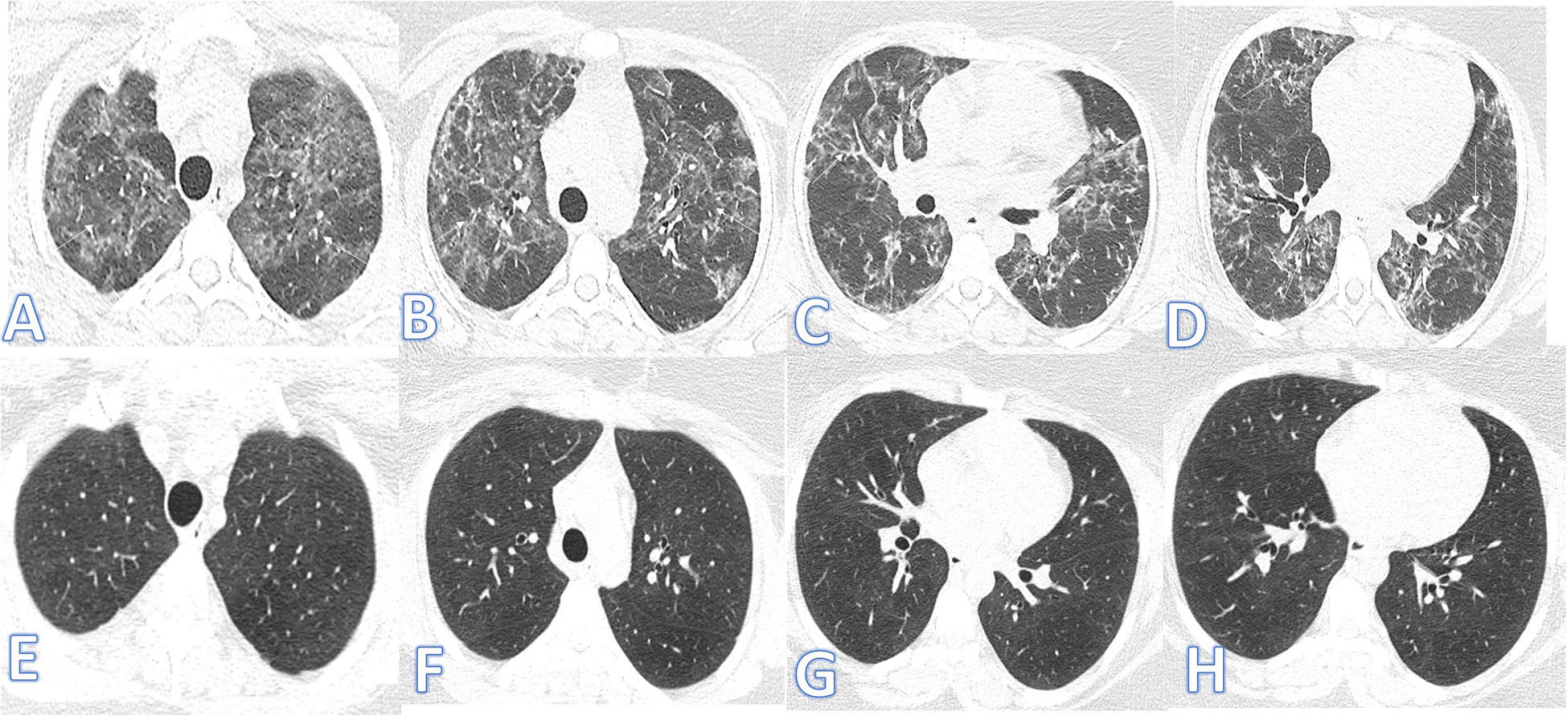
Thirty-year-old female: she has no previous medical illness; CT severity score 15; consolidation/crazy-paving score 10. No ICU admission; laboratory results showed normal lymphocyte and serum ferritin, high CRP, d-dimer. Steroid was given; length of stay during hospitalization is 14 days. Peak CT during admission (**a**–**d**) showed bilateral central and peripheral GGO mainly in the upper lobes, associated with lung fibrosis with parenchymal bands and coarse reticular pattern (arrows). Follow-up CT 2 months after discharge (**e**–**h**) showed total resolution of the previous lung changes

**Fig. 6:**
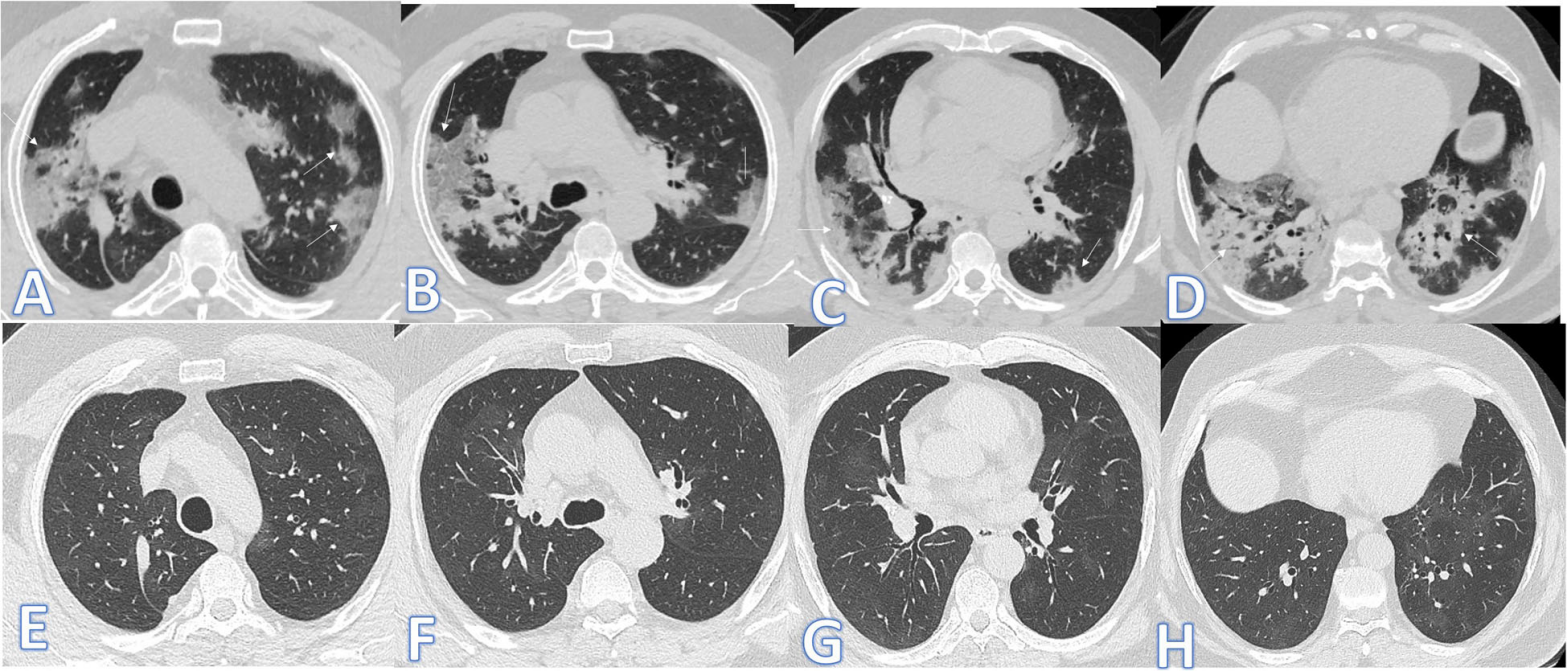
Twenty-five-year-old male: he has no previous medical illness: CT severity score 14; consolidation/crazy-paving score 12. No ICU admission; laboratory results show lymphopenia, high CRP, d-dimer, and serum ferritin. Steroid was given; length of stay during hospitalization is10 days. Peak CT during admission (**a**–**d**) showed bilateral central and peripheral consolidation patches mainly in the lower lobes with GGO and a crazy-paving pattern (arrows). Follow-up CT 2 months after discharge (**e**–**h**) showed total resolution of the previous lung changes

The demographic and clinical data of the two groups are comparatively presented in Table 3; there was a statistically significant difference between both groups as regards patient’s age (Figs. 4, 5, and 6) with no statistically significant difference regarding sex. Patients in the fibrotic group were older than those in the non-fibrotic group (mean age, 58.81±14.82 vs 49.26±13.36 years) (*p* <0.001).

**Table 3:** Comparison between COVID-19 patients with and without evidence of fibrosis. Non-fibrotic group (*n* =109); fibrotic group (*n* =101)

	The studied group, *N* = 210		Test	*p* value
	Non fibrotic, *N* = 109	Fibrotic, *N* = 101		
**Age (years)**				
Mean ±SD	49.26±13.36	58.81±14.82	*t* test 4.91	<0.001
Range	18–76	24–94		
**Sex**				
Male	76 (69.7%)	73 (72.3%)	*X*^2^ 0.17	0.65
Female	33 (30.3%)	28 (27.7%)		
**Severity score**				
Mean ±SD	7.55±3.32	15.20±3.34	*U* 11.09	<0.001
Range	1–14	9–20		
**Consolidation/crazy-paving score**				
Mean ±SD	5.42±3.55	12.63±4.22	*U* 10.06	<0.001
Range	0–15	5–20		
**Lymphopenia**	64 (58.7%)	79 (78.2%)	*X*^2^ 9.2	0.002
**Elevated high-sensitivity troponin**	17 (15.6%)	28 (27.7%)	*X*^2^ 4.6	0.03
**High ferritin levels**	62 (56.9%)	81 (80.2%)	*X*^2^ 13.1	<0.001
**Elevated CRP**	90 (82.6%)	95 (94.1%)	*X*^2^ 6.6	0.01
**Elevated** **d-dimer**	87 (79.8%)	96 (95.0%)	*X*^2^ 10.9	0.001
**Length of hospital stay**				
Mean ±SD	8.56±7.03	23.26±20.89	*U* 8.26	<0.001
Range	1–37	2–170		
**ICU admission**				
No	102 (93.6%)	56 (55.4%)	*X*^2^ 40.9	<0.001
Yes	7 (6.4%)	45 (44.6%)		
**Steroid**				
No	27 (24.8%)	7 (6.9%)	*X*^2^ 12.3	<0.001
Yes	82 (75.2%)	94 (93.1%)

**Fig. 7:**
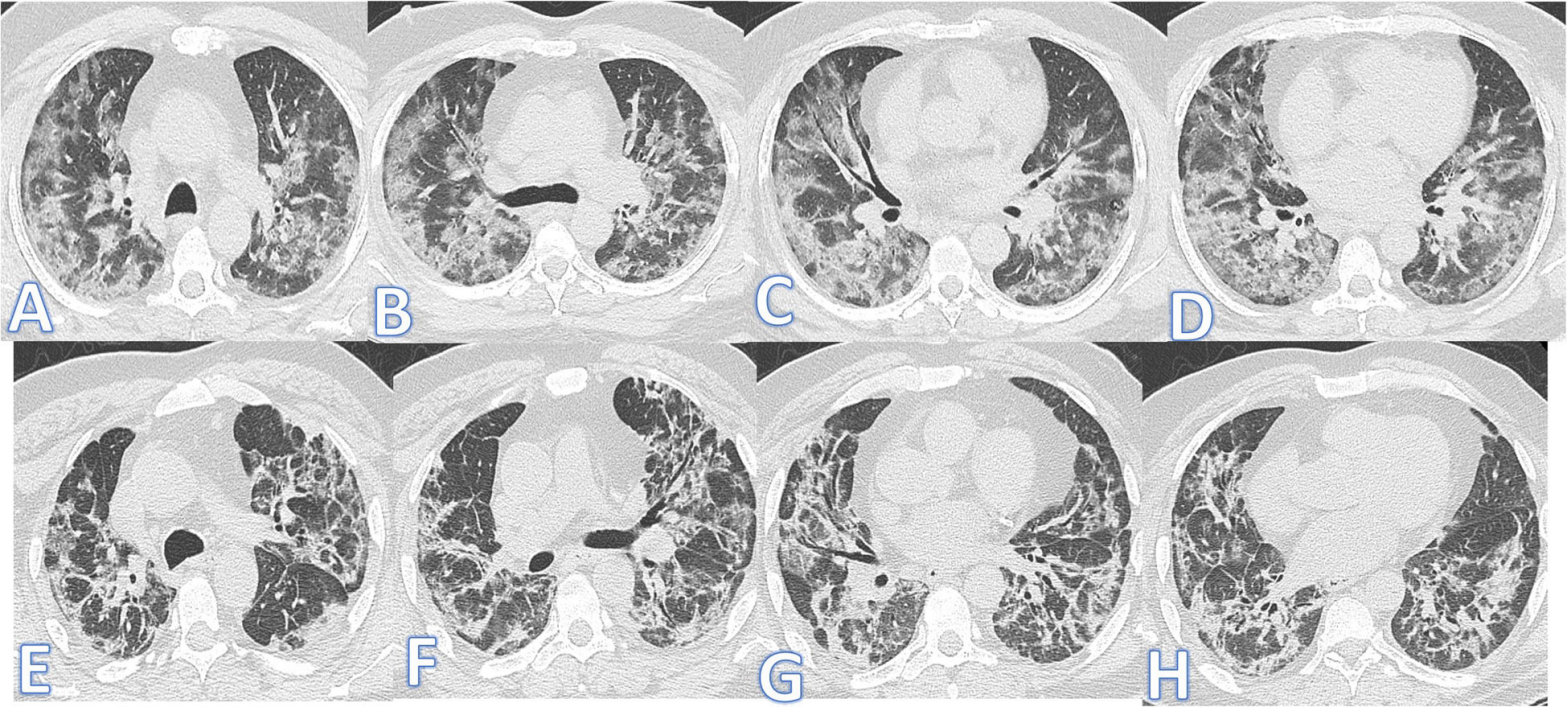
Fifty-five-year-old male: he has DM, HTN, and IHD; peak CT severity score 16; consolidation/crazy-paving score 14. He was admitted to the ICU; laboratory results showed lymphopenia, high CRP, d-dimer, normal serum ferritin, and sensitivity troponin. Steroid was given; length of stay during hospitalization is 30days. Peak CT during admission (**a**–**d**) showed bilateral consolidation patches more peripheral and at the lower lobes with a crazy-paving appearance. Follow-up CT 3weeks after discharge (**e**–**h**) showed lung fibrosis with parenchymal bands, coarse reticular pattern, irregular interface, and mild traction bronchiectasis at the anterior segments of both upper lobes and the medial segment of the right middle lobe

**Fig. 8:**
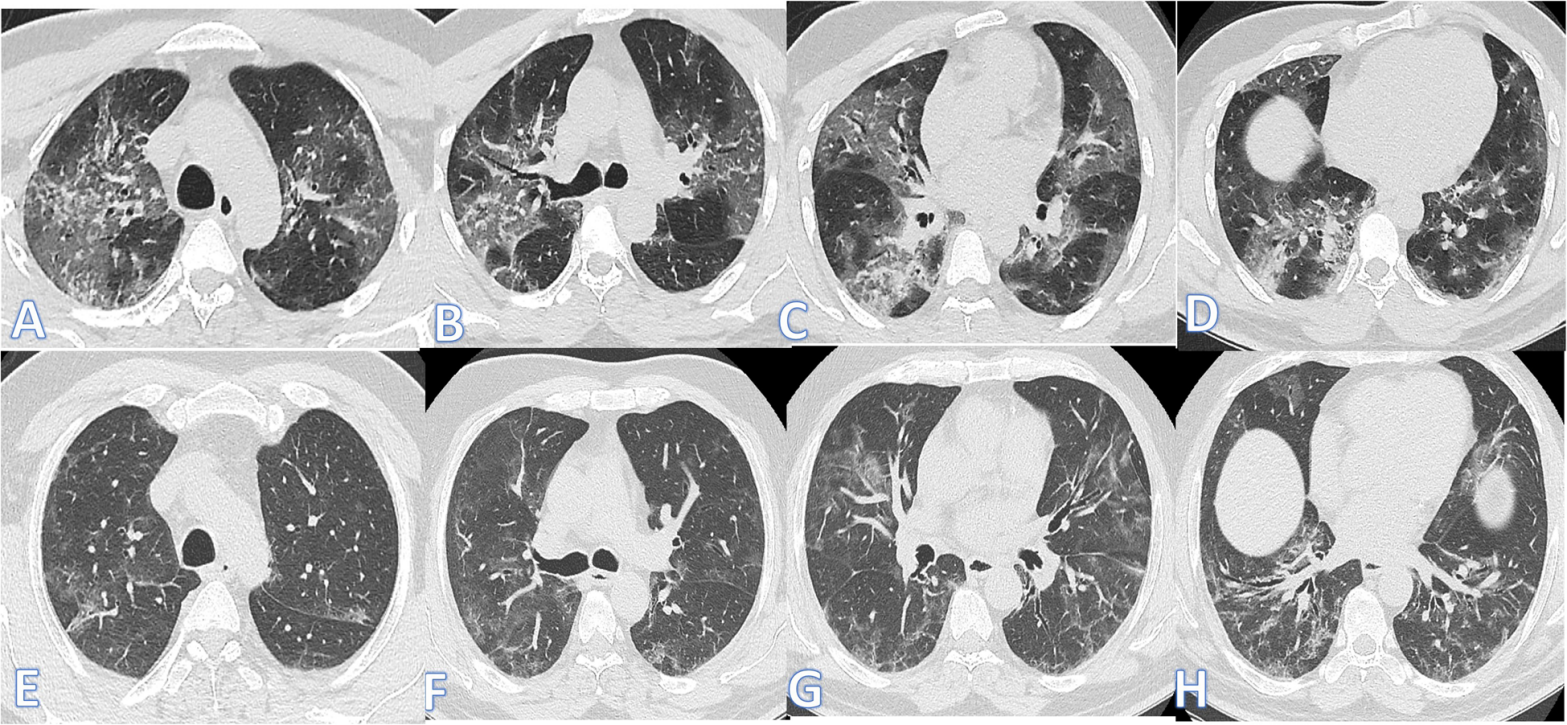
Thirty-nine-year-old male: he has DM and HTN; peak CT severity score 17; consolidation/crazy-paving score 13. He was admitted to the ICU; laboratory results showed lymphopenia, high CRP, d-dimer, and serum ferritin. Steroid was given; length of stay during hospitalization is 20 days. Peak CT during admission (**a**–**d**) showed bilateral GGO with mild interstitial thickening and basal consolidation patches. Follow-up CT 2months after discharge (**e**–**h**) showed lung fibrosis with parenchymal bands, coarse reticular pattern, and mild bilateral traction bronchiectasis

Regarding ICU admission (Figs. 7 and 8), steroid therapy, and length of stay (LOS) in the hospital, there was also a statistically significant difference between both groups (*p* ˂0.001). The LOS in the fibrotic group was longer than that in the non-fibrotic group (23.26±20.89 vs 8.56±7.03 days). The percentage of ICU admission in the fibrotic group was higher than in the non-fibrotic group (44.6% vs 6.6%).

In comparison with both groups in laboratory studies including lowest lymphocyte level, C-reactive protein (CRP) level, serum ferritin, high-sensitivity troponin, and d-dimer, there was a statistically significant difference between both groups (*p* ˂0.001) with higher serum levels detected among the fibrotic group.

**Fig. 9:**
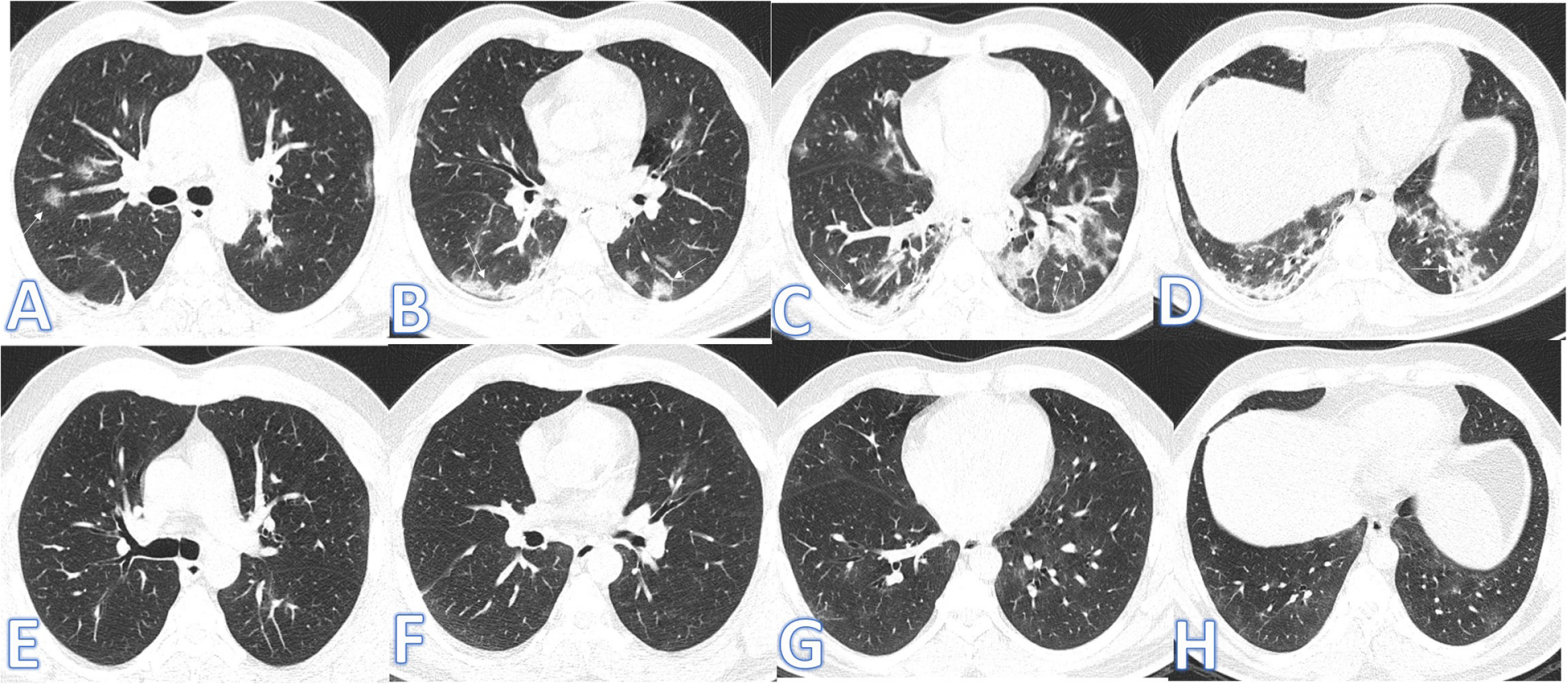
Thirty-eight-year-old male: he has no previous medical illness; CT severity score 7, consolidation/crazy-paving score 4. No ICU admission; laboratory results showed normal lymphocytes, serum ferritin, and d-dimer and high CRP and d-dimer. Steroid was not given; length of stay during hospitalization is 2days. Peak CT during admission (**a**–**d**) showed bilateral peripheral consolidation patches mainly in the lower lobes with rounded GGO (arrows). Follow-up CT 1month after discharge (**e**–**h**) showed total resolution of the previous lung changes

As regards both CT severity score and consolidation/crazy-paving score calculated from the initial CT during hospitalization, there was a statistically significant difference between the two groups (*p* ˂0.001) with higher values observed among the fibrotic group compared to the non-fibrotic group (Fig. 9).

Based on these variables, a further multivariate analysis using the forward method was performed, and it was found that the age of the patients, initial CT severity score, consolidation/crazy-paving score, and ICU admission were independent risk factors associated with the presence of post-COVID-19 fibrosis (*p*<0.05, Table 4).

**Table 4:** Multivariate regression analysis for independent risk factors for prediction of post-COVID-19 fibrosis

	SE	Wald *X*^2^	*p* value	Odds ratio	95% CI
**Age (years)**	0.02	9.39	0.002	3.37	0.76–14.55
**Severity score**	0.12	8.95	0.003	2.38	1.18–4.41
**Consolidation/crazy-paving score**	0.11	1.93	0.04	1.91	0.63–4.35
**Lymphocytes**	0.53	0.49	0.49	0.70	0.25–1.96
**High-sensitivity troponin**	0.66	0.29	0.59	1.18	0.90–1.89
**Ferritin**	0.52	0.40	0.84	0.90	0.35–2.42
**CRP**	0.06	0.67	0.41	1.12	0.38–9.19
**d-dimer**	0.0	2.59	0.05	1.98	1.01–10.19
**Length of hospital stay**	0.01	0.02	0.90	1.0	0.97–1.13
**ICU admission**	0.69	7.82	0.005	6.77	1.77–25.88
**Steroid**	0.74	0.16	0.69	1.01	0.24–4.28

**Fig. 10:**
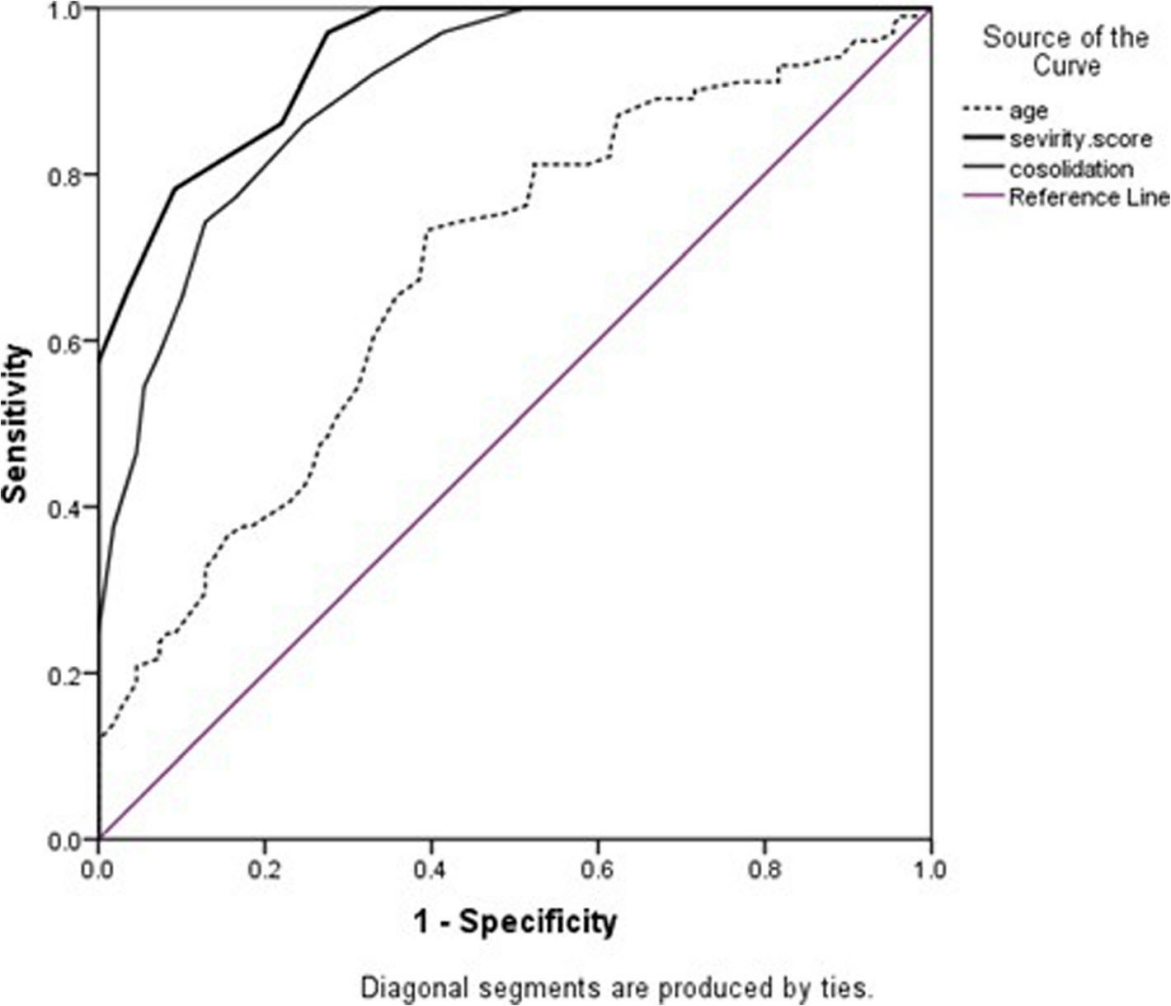
ROC curve for age, chest CT severity, and consolidation/crazy-paving scores as a predictor for post-COVID-19 fibrosis to detect the sensitivity and specificity of these factors

Analysis of the ROC curve for independent factors for the prediction of post-COVID-19 fibrosis is shown in Fig. 10 and Table 5; the highest area under the curve (AUC) of 0.94 was for chest CT severity score showing a sensitivity of 86.1%, a specificity of 78% and accuracy of 81.9% at a cutoff point of 10.5.

**Table 5:** Accuracy of independent factors for prediction of fibrosis

	Age	Severity score	Consolidation	ICU admission
**AUC**	0.68	0.94	0.90	–
***p*** **value**	<0.001	<0.001	<0.001	–
**95% CI**	0.61–0.76	0.92–0.97	0.86–0.94	–
**Cutoff point**	51.5	10.5	7.5	–
**Sensitivity**	73.3%	86.1%	86.1%	44.6%
**Specificity**	59.6%	78%	75.2%	93.6%
**PPV**	62.7%	78.4%	76.3%	86.5%
**NPV**	70.7%	85.9%	85.4%	64.6%
**Accuracy**	66.2%	81.9%	80.5%	70.0%

## Discussion

Over the past months, HRCT had participated effectively in the diagnosis of COVID-19 and assessment of disease severity. The major imaging features of COVID-19 pneumonia have been discussed in detail in many publications [20]. However, the post-recovery outcome of the disease and its long-term effects on lung parenchyma remain unanswered questions.

The previous experience with SARS and MERS showed that follow-up CT is recommended in individuals recovering from COVID-19 to evaluate which group of patients is more likely to develop pulmonary fibrosis [21].

In this study, we aimed to evaluate the radiological findings on follow-up HRCT of patients recovered from COVID-19 and identify the possibility of pulmonary fibrosis in discharged patients after treatment.

At the current study, patients were classified into two groups according to the presence of fibrosis on follow-up CT after discharge: “fibrotic group” (with evident fibrosis) and “non-fibrotic group” (without evident fibrosis). Out of 210 patients, 101 patients (48.1%) showed fibrosis on follow-up CT while 109 patients (51.9%) had no evident fibrosis.

Our results showed a statistically significant difference between the two groups regarding the patient’s age where patients in the fibrotic group were significantly older than those in the non-fibrotic group indicating that elderly patients are more liable to develop fibrosis following COVID-19.

In line with our study, Yu et al. [22] found that patients who developed fibrosis on follow-up after discharge were older than those without fibrosis, suggesting that fibrosis was more common in elderly patients, similar to SARS. They stated that patients with fibrosis had a longer length of stay in the hospital with a higher rate of ICU admission and a higher level of CRP than those without fibrosis; they also reported that patient with fibrosis had received more pulsed steroid therapy and antiviral therapy and for a longer period compared with patients without fibrosis; thus, these clinical parameters during acute disease may help in the prediction of the risk of developing pulmonary fibrosis after discharge.

Similarly, our results showed that patients in the fibrotic group showed a longer length of stay in the hospital and a longer duration of ICU admission and steroid therapy (*p* ˂0.001). In comparison with both groups regarding the laboratory results including the lowest lymphocytic level, CRP level, serum ferritin, high-sensitivity troponin, and d-dimer levels, there were statistically significant differences between both groups (*p* ˂0.001) with higher serum levels detected among patients in the fibrotic group suggesting that higher inflammatory markers are more associated with developing fibrosis.

Shi et al. [23] found that on COVID-19 patients, cardiac troponin is a prognostic marker with a strong association with mortality observed in the currently available reports of patients hospitalized with COVID-19, with some evidence suggesting cardiac troponin T/I even as an independent predictor of mortality.

At the current study, higher values for CT severity score and consolidation/crazy-paving score on the initial CT were found in the fibrotic group compared to the non-fibrotic group suggesting that patients with severe disease are more liable to fibrosis after discharge.

In the same context, Wei et al. [24] included 59 patients who were treated for COVID-19 pneumonia in a multi-center study and had a follow-up CT within 1 month after being discharged from four hospitals in China; their study showed that 39% of patients had residual fibrosis while 61% had no evidence of fibrosis on HRCT. They found that elderly patients had a higher chance of developing fibrosis, and patients who developed fibrosis had a higher CT score, higher ICU admission, and higher C-reactive protein. They also cited previous work by Antonio et al. [19] in studying severe cases of SARS who stated that early lung fibrosis rate reaches as high as 62% in SARS. COVID-19 showed a lower rate of fibrosis than SARS. In addition, SARS causes severe lung parenchymal damage than COVID-19.

Likewise, Das et al. [25] stated that 33% of patients with MERS show lung fibrosis on follow-up CT. These patients were older in age, had a longer duration of ICU admission, and had more severe lung involvement in the acute stage of the disease.

Additionally, we performed multivariate analysis for predictors of post-COVID-19 fibrosis, and we found that among these previous significant variables, patient’s age, initial CT severity score, consolidation/crazy-paving score, and ICU admission were independent risk factors associated with post-COVID-19 fibrosis (*p*<0.05, Table 4). Further analysis of the ROC curve for independent factors was done and showed the highest AUC for chest CT severity score reflecting a good predictive value for post-COVID-19 fibrosis with a sensitivity of 86.1% and a specificity of 78% at a cutoff point of 10.5.

Yu et al. [22] compared the imaging features between a group of patients with fibrosis and without fibrosis regarding the initial CT; they found that more patients in the fibrosis group had interstitial thickening, coarse reticulations, and subpleural/parenchymal bands on the initial CT. Also, more lung segments were involved on the initial CT in patients in the fibrosis group than in the non-fibrosis group. They suggested that these findings on the initial CT as interstitial thickening, reticulations, and parenchymal bands might be predictors of pulmonary fibrosis in recovered patients since they have similar pathogenesis.

This was matching with our results that showed more lung segment affection on the initial CT in the fibrotic group compared to the non-fibrotic group (*p*<0.001). Pure GGO was statistically higher in the non-fibrotic group, while pure consolidation or GGO with consolidation, crazy paving, air bronchogram, and fibrotic changes were significantly higher in the fibrotic groups (*p*<0.001).

At the current study, follow-up CT after discharge was performed at an average time of 41.5 days (range 20–65 days) after discharge, and it showed persistent parenchymal abnormalities with fibrotic changes in 48.1% of patients while 51.9% of patients had no residual parenchymal changes or fibrosis.

These results were in accordance with the findings reported by Liu et al. [26] who performed a 3-week follow-up study to determine the cumulative percentage of complete radiological resolution of pulmonary changes in discharged patients recovering from COVID-19. They stated that in 53% of patients, the pulmonary changes were completely absorbed at the 3rd week after discharge, reflecting that pulmonary damage induced by COVID-19 could be potentially repaired without permanent sequelae. However, more than 40% of patients at the 3rd week radiological follow-up showed residual parenchymal abnormalities, including GGO and fibrous parenchymal bands. In their study, younger age was associated with more complete radiological resolution.

Zaho et al. [27] in a multi-center cohort study found that residual pulmonary abnormalities and abnormal CT scores persisted for 3 months after discharge in 70.91% of COVID-19 survivors. Bilateral lung involvement was still found in 23.6% of patients and in about half of patients (54.55%); 1–3 lung segments were involved. Typical parenchymal features were almost resolved, but evidence of fibrosis was observed. The most common CT feature found on the latest follow-up was interstitial thickening (27.27%).

The current study has limitations to be acknowledged. First, small sample size relative to the disease burden. Therefore, studies in larger samples should be considered. Second, fibrosis was not confirmed by histopathology even though imaging manifestations were diagnostic. Third, follow-up time was relatively short, and it is unknown whether these pulmonary changes will resolve on further follow-up or permanently remain, so long-term follow-up studies are recommended for further research.

## Conclusion

The residual pulmonary fibrosis in COVID-19 survivors after discharge depends on many factors with patient’s age, CT severity, consolidation/crazy-paving scores, and ICU admission were independent risk factors associated with the presence of post-COVID-19 fibrosis.

## Data Availability

All data and materials are available.

## Abbreviation

HRCT: High-resolution computed tomography
SARS-CoV: Severe acute respiratory syndrome coronavirus
MERS-CoV: Middle East respiratory syndrome coronavirus
ARDS: Acute respiratory distress syndrome
ICU: Intensive care admission
LOS: Length of stay
RT-PCR: Reverse transcriptase polymerase chain reaction
CRP: C-reactive protein
GGO: Ground glass opacity
CT: Computerized tomography
SPSS: Statistical Package for Social Science
ROC: Receiver operating characteristic
AUC: Area under the curve
